# PReS-FINAL-2096: Herpes virus infections in patients with juvenile idiopathic arthritis (JIA) treated with etanercept

**DOI:** 10.1186/1546-0096-11-S2-P108

**Published:** 2013-12-05

**Authors:** R Nicolai, E Cortis, L Ravà, C Bracaglia, M Pardeo, A Insalaco, PS Buonuomo, F De Benedetti, AE Tozzi

**Affiliations:** 1 Division of Rheumatology, Bambino Gesù Children's Hospital, Rome, Italy; 2 Department of Pediatrics, Santa Maria della Stella Hospital, Orvieto, Italy; 3 Division of Epidemiology, Bambino Gesù Children's Hospital, Rome, Italy; 4 Division of Rare Diseases, Bambino Gesù Children's Hospital, Rome, Italy

## Introduction

TNF-α is involved in regulation of herpes virus replication and dissemination, and varicella, herpes zoster and herpes labialis have been observed as adverse events during clinical trials with and in national registries for the use of etanercept in JIA, in some cases resulting in serious adverse events (saes).

## Objectives

The aim of this study was to obtain incidence data of primary varicella, herpes zoster and herpes labialis in patients with JIA and to compare incidence rates of patients receiving etanercept with those of patients treated with methotrexate (MTX) alone.

## Methods

Eighty-five (85) patients with JIA receiving etanercept in combination with MTX (etanercept/MTX) and 71 patients receiving MTX alone were enrolled at the Division of Rheumatology, Bambino Gesù Children's Hospital, Rome, Italy. Incidence rates were calculated as the number of varicella cases, herpes zoster episodes and herpes labialis episodes per patient-years of follow-up (under specific treatment).

## Results

Mean treatment exposure was 2.17+/-1.57 years for etanercept/MTX (184.85 patient-years of total follow-up under treatment) and 2.80+/- 1.81 years for MTX (199.42 patient-years). Crude incidence rates for herpes labialis were significantly higher among patients receiving etanercept/MTX (70 episodes in 15 patients) compared to MTX alone (48 episodes in 10 patients)(0.37 vs 0.24 per patient-year, p = 0.015). We found no significant difference in crude incidence rates between etanercept/MTX versus MTX treatment group for primary varicella infection (0.06 vs 0.05 per patient-year, P > 0.05) and herpes zoster (0.01 vs 0.005 per patient-year, P > 0.05). All patients who developed varicella received antiviral treatment. No complicated varicella infections requiring hospitalization and no cases of multidermatomal zoster, herpes zoster ophthalmicus or post-herpetic neuralgia were reported. In the univariate Kaplan-Meier analysis, there was a statistically significant association only between herpes labialis and polyarticular involvement (P: 0.024); multivariate Cox regression analysis did not confirm this association.

## Conclusion

In our study population the incidence rate for herpes labialis recurrences was significantly higher in the etanercept/MTX group. Patients with polyarticular JIA more likely have highly active disease; disease activity may affect cellular-immunity, which could explain the increased herpes labialis risk observed in this subgroup of JIA patients. The main limitations of our study are the size of the study population and the observational nature of the study.

## Disclosure of interest

None declared.

